# Transcriptome Analysis of the *Octopus vulgaris* Central Nervous System

**DOI:** 10.1371/journal.pone.0040320

**Published:** 2012-06-29

**Authors:** Xiang Zhang, Yong Mao, Zixia Huang, Meng Qu, Jun Chen, Shaoxiong Ding, Jingni Hong, Tiantian Sun

**Affiliations:** 1 State Key Laboratory of Marine Environmental Science, Xiamen University, Xiamen, China; 2 The Laboratory of Marine Biodiversity and Global Change, Xiamen University, Xiamen, China; 3 College of Ocean and Earth Sciences, Xiamen University, Xiamen, China; Kyushu Institute of Technology, Japan

## Abstract

**Background:**

Cephalopoda are a class of Mollusca species found in all the world's oceans. They are an important model organism in neurobiology. Unfortunately, the lack of neuronal molecular sequences, such as ESTs, transcriptomic or genomic information, has limited the development of molecular neurobiology research in this unique model organism.

**Results:**

With high-throughput Illumina Solexa sequencing technology, we have generated 59,859 high quality sequences from 12,918,391 paired-end reads. Using BLASTx/BLASTn, 12,227 contigs have blast hits in the Swissprot, NR protein database and NT nucleotide database with E-value cutoff 1e^−5^. The comparison between the *Octopus vulgaris* central nervous system (CNS) library and the *Aplysia californica*/*Lymnaea stagnalis* CNS ESTs library yielded 5.93%/13.45% of *O. vulgaris* sequences with significant matches (1e^−5^) using BLASTn/tBLASTx. Meanwhile the hit percentage of the recently published *Schistocerca gregaria*, *Tilapia* or *Hirudo medicinalis* CNS library to the *O. vulgaris* CNS library is 21.03%–46.19%. We constructed the Phylogenetic tree using two genes related to CNS function, Synaptotagmin-7 and Synaptophysin. Lastly, we demonstrated that *O. vulgaris* may have a vertebrate-like Blood-Brain Barrier based on bioinformatic analysis.

**Conclusion:**

This study provides a mass of molecular information that will contribute to further molecular biology research on *O. vulgaris*. In our presentation of the first CNS transcriptome analysis of *O. vulgaris*, we hope to accelerate the study of functional molecular neurobiology and comparative evolutionary biology.

## Introduction

Cephalopods are a group of unique Mollusk species that evolved in the Late Cambrian. There are around 800 extant species in Cephalopoda, none of which live in fresh water. Cephalopods possess many primitive traits of invertebrates (the genital chamber is connected with the body cavity; there is no kidney in the early embryonic development period, and the genital duct derives from the coelomic duct), but also exhibit more advanced features such as the vertebrate-like eye, no larval phase in ontogenesis, and a highly centralized nervous system. Cephalopods' hybrid evolutionary characteristics suggest that the Cephalopoda are a special branch of Mollusca. The Cephalopods' nervous system is the most complex of the invertebrates. This has led to its use in research. For example, the squid giant axon was used to prove the mechanism of action potentials by John Carew Eccles, Alan Lloyd Hodgkin and Andrew Fielding Huxley, for which they won the 1963 Nobel Prize. Recently, the use of Cephalopods in neurobiology research has been limited. In contrast, molecular neurobiology research using high-throughput transcriptome sequencing technology in other Mollusk species has been increasing rapidly. Leonid L Moroz established a central nervous system (CNS) database of *Aplysia californica* for gene discovery, expression profiling, and characterization of signaling pathways in 2006 [1]. In 2009, Z.P. Feng and Z. Zhang provided a *Lymnaea stagnalis* CNS EST database and used it to illustrate the substantial genetic diversity within *A. californica* [2].

Through the evolutionary process, the structure and function of the central nervous system has become more complex. Some of these adaptations are important for physical protection of the CNS. The Blood-Brain Barrier is one example. It has been demonstrated to be an important structure for maintaining the basic stability of the internal environment. In 1885, Paul Ehrlich found that vital dyes can stain all mammalian organs except the brain. Edwin Goldmann demonstrated that if dye were injected into the cerebro-spinal fluid, then the central nervous system (CNS) would be stained, but no other organs. These results suggest that there exists a barrier between the CNS and the peripheral organs. Blood-Brain Barrier function is controlled by the brain's microvascular endothelial cells (BMVEC), basement membrane, and four different types of neighboring glial cells such as astrocytes, perivascular pericytes and microglia. Neurons are also important for the function of the CNS and BBB. The brain's microvascular endothelial cells (EC), and cellular connection between the BMVEC were demonstrated to play a key role in BBB function [3]. EC supply high electric impedance, specific receptors and transporters, while the specialized junctions eliminate gaps between EC and prevent free substances interchange between the blood and brain parenchymal space [4]–[7]. Research related to the BBB are not only in vertebrates; in invertebrates such as Drosophila there also exists a barrier system to protect the CNS, albeit a different one [8]. It seems that the barrier system was necessary in organisms with a more active lifestyle. Unlike other Mollusk species, Cephalopods live a more active life. Cephalopods also have a CNS structure and closed vascular system similar to vertebrates. As of yet, there is no direct evidence that Cephalopods have a vertebrate-like BBB. Discovery of a vertebrate-like BBB would accelerate the understanding of BBB structure, function and origin.

*O. vulgaris* is one of the commercially valuable benthic species among Cephalopods. It is widely distributed in the Pacific, Atlantic and Indian Oceans, as well as the Mediterranean Sea. It belongs to the phylum Mollusca (Cephalopoda, Dibranchiata, Octopoda, Octopodidae). The economic value and wide distribution make it easy to sample, and aid in its use as a model organism. In recent years, most *O. vulgaris* research has been focused on larval growth [9]–[12], physiology [13]–[16], ecological toxicology [17]–[22] and fisheries [23]–[26]. Due to a lack of molecular sequence information, the development of molecular biology research in *O. vulgaris* is limited.

In this study, we dissect a complete adult *O. vulgaris* for CNS tissue to prepare the first non-normalized cDNA library. We use high-throughput Illumina Solexa sequencing technology to acquire a mass of CNS molecular information about the Cephalopod *O. vulgaris*. After assembly, gene name annotation and GO/KEGG annotation, this transcriptome data was used to develop a better understanding of *O. vulgaris*, such as a molecular foundation of its biological characteristics and phylogenetic analysis of *O. vulgaris* versus other published CNS transcriptome data. It was also a great resource for mining information about genes related to the CNS and vertebrate-like Blood-Brain Barrier. We present the first CNS transcriptome analysis of Cephalopoda species *O. vulgaris* aimed to accelerate the study of functional molecular neurobiology and comparative evolutionary biology in Cephalopoda.

## Results and Discussion

### 1. Illumina sequencing and sequence assembly

**Figure 1:**
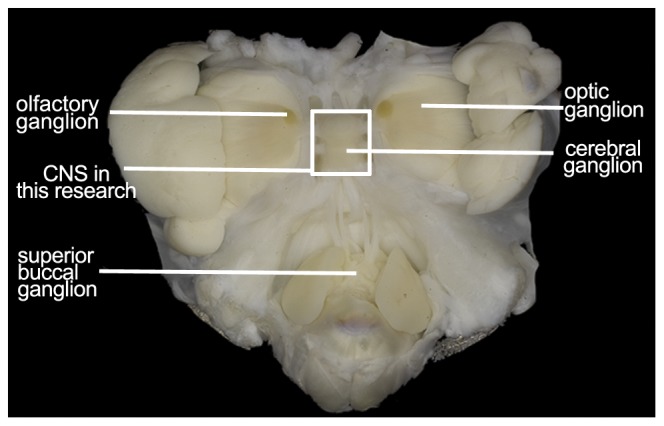
Detection of the central nervous system (CNS) of *O. vulgaris*.

**Figure 2:**
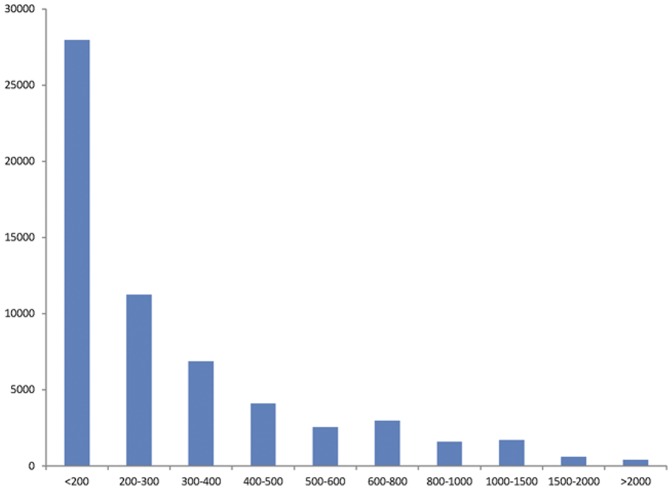
Length distribution of contigs obtained from *O. vulgaris* central nervous system (CNS) transcriptome library.

We dissected a complete adult *O. vulgaris* for CNS, including cerebral ganglion, visceral ganglion, pedal ganglion, and part of the peripheral nervous system (Figure 1). The non-normalized cDNA library is constructed following the manufacturer's instructions (Illumina). High-throughput SOLEXA paired-end sequencing yielded a total of 13,753,396 reads with length of 90 bp and Q20 percentage equal 96.38%. 834,465 sequences were removed because of low quality. These high-quality sequences were assembled with VELVET [27] and a summary table for assembly was deposited to Table S1. For best assembly results, four important parameters were tested, including K-mers, coverage, minimum contigs length, and numbers of contigs. 12,918,931 reads were assembled into 59,859 contigs with a maximum length of 8,970 bp and the N50 length of 450 bp. Then the CD-HIT program [28] was performed and the results showed that only 48 (<0.1%) sequences had significant similarities (>98% identity and >95% coverage) against other sequences within the dataset, indicating the assembly was accomplished and high quality. The length distribution for all contigs is presented in Figure 2. Of these, a total of 31,315 open reading frames (ORFs≥50 aa) have been detected (Figure S1).

### 2. BLASTX searches in Swissprot and NR protein database

Contig gene name annotation was archived through BLASTx and BLASTn searches against the Swissprot,NR (NCBI non-redundant protein database), and NT (NCBI nucleotide sequences database). Using Perl, the description of the most relevant hits with E-value less than 1e^−5^ were assigned to the query sequences. This search revealed that only 10,412 (17.39%) and 1,815 (3.03%) sequences have a significance blast hit (1e^−5^). The reason why most of the sequences (79.58%) didn't have a significant blast hit is probably caused by potentially novel genes and a lack of molecular information of closely related species in Cephalopods.

Table 1 is a list of the 20 sequences, sorted by read number, which revealed the most expressed proteins or peptides. All sequences with high read number have been annotated. This result may illustrate the vivid biological characteristics of the CNS transcriptome, including several neurohormones, neuro-consisted proteins and stress-response proteins. Some proteins may involve features specific to *O. vulgaris*, such as fatty acid-binding proteins and apolopophorins which reflect the strong lipid metabolism of *O. vulgaris*, and retinol dehydrogenase which convert retinol into retinal to maintain vision.

**Table 1:** List of top 20 sequences sorted by reads.

Gene description	Reads Number	Contig length (bp)
Collagen alpha-1(II) chain	45238.7	1273
Collagen alpha-1(I) chain	41466.5	911
70 kDa neurofilament protein	38630.7	1696
Actin-2	32603.0	510
Fatty acid-binding protein	27441.6	225
Retinol dehydrogenase 12	26218.4	1232
Tubulin beta-4 chain	26149.6	750
Apolipophorins	25684.2	2644
Collagen alpha-1(II) chain	24065.9	306
Orcokinin peptides type A	23617.8	721
14-3-3 protein epsilon	22279.9	1177
Elongation factor 1-alpha	21795.1	175
Collagen alpha-1(XIII) chain	21554.9	1208
Elongation factor 2	21005.5	987
Tubulin beta-2C chain	20963.0	498
Heat shock cognate 71 kDa protein	19960.3	486
Glycogen phosphorylase, muscle	19659.6	2083
Tubulin beta chain	19452.1	655
Heterogeneous nuclear ribonucleoprotein U-like protein 1	18672.2	5343
2-phosphoglycerate dehydratase	18341.2	1473

### 3. Functional annotation based on GO and KEGG analysis

**Figure 3:**
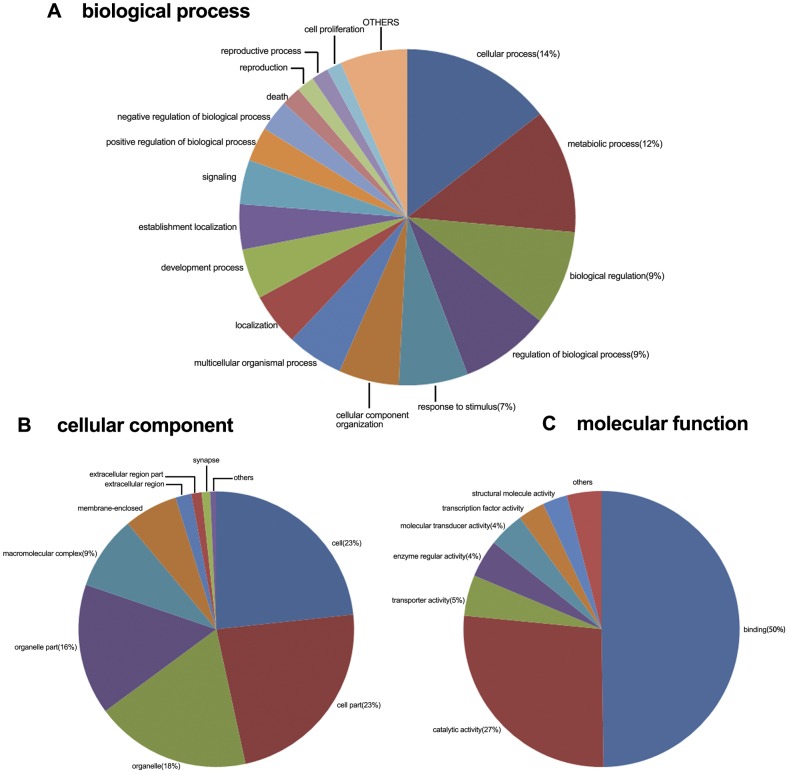
Distribution of second level GO annotation in three categories.

In order to determine the functions of these sequences, 12,227 genes were selected to annotate with the GO database by Gominer [29]. Figure 3 shows the gene number of GO functional annotation analysis in level 2.

By selecting the defining GO term with keywords “neuro” and “nervous”, more than 50 GO terms are involved and their matching genes ranged from 1 to 338. The largest GO categorie related to CNS function is nervous system developments (GO index: 0007399). Table 2 presents the number of genes matched in 15 GO terms that are relevant to neuronal functions and have the largest numbers matching, demonstrating our transcript database has a great number of neural sequences that will provide a great resource for further research of the development, function and regulatory mechanisms of the *O. vulgaris* central nervous system.

**Table 2:** GO categories with highest number of sequences corresponding to CNS function.

GO term	GO Index	Total
Nervous system development	0007399	338
Neurogenesis	0022008	216
Generation of neurons	0048699	203
Neuron differentiation	0030182	193
Neurological process	0050877	175
Neuron development	0048666	160
Neuron projection development	0031175	138
Central nervous system development	0007417	132
Neuron projection	0043005	117
Neuron projection morphogenesis	0048812	117
Neuron morphogenesis involved in differentiation	0048667	117
Transmission of nerve impulse	0019226	116
Synaptic transmission	0007268	103
Axonogenesis	0007409	100
Regulation of nervous system development	0051960	77

KEGG (Kyoto Encyclopedia of Genes and Genomes) is a pathway-based categorization of orthologous genes that provides useful information for predicting functional profiles of genes [30]. To further demonstrate the biological pathways that are active in the CNS of *O. vulgaris*, 3,825 genes were mapped into the signaling pathways in the KEGG.

### 4. Comparisons with the previously published Mollusc species *A. californica* and *L. stagnalis* CNS database

The published CNS transcriptome database of *A. californica* [2] and *L. stagnalis* [1] give us an opportunity to understand the diversity between *O. vulgaris* and other Mollusk species. Tables 3 and 4 provides the data of BLASTn or tBLASTx hits between *O. vulgaris* and *L. stagnalis* (or *A. californica*). In the comparison between *O. vulgaris* and *L. stagnalis* ESTs (10,375 ESTs which assemble into 7,712 unique sequences) by BLASTn and tBLASTx, at E-value cutoff of 1e^−5^, about 7.83% (BLASTn) and 26.53% (tBLASTx) of *L. stagnalis* cDNAs have hits in *O. vulgaris* CNS library. In the mean time, approximately 1.03% (BLASTn) and 5.77% (tBLASTx) of the sequences in *O. vulgaris* CNS library have a hit in *L. stagnalis* cDNAs. Considering these two databases are constructed from CNS tissues, the main cause of low percentages of blast hits could be the difference in their genetic codes. In addition, these results may be explained by: 1) the sequences below 200 bp are unlikely to have a blast hit 2) it is possible that some genes exist in both species, but have expression levels below the detection limit of the transcriptome studies, and 3) differing expression profiles between different species or developmental stages.

**Table 3:** Comparison between the *O. vulgaris* CNS dataset and *L. stagnalis* CNS dataset using BLASTn and tBLASTx.

Most significance Blast E-value	Numbers of *L. stagnalis* ESTs having a blast hit in *O. vulgaris*		Numbers of *O. vulgaris* cDNAs having a blast hit in *L. stagnalis* ESTs	
	Blastn	TblastX	Blastn	TblastX
E<1 e^−20^	231	1454	178	1617
1 e^−20^<E<1 e^−15^	78	136	65	300
1 e^−15^<E<1 e^−10^	101	199	113	486
1 e^−10^<E<1 e^−5^	194	258	259	1053
1 e^−5^<E<1	6009	4857	51316	46719
Total (E<1 e^−5^)	2145		3551(3543)	

The number in each bracket shows the number of genes after removed redundant sequences.

**Table 4:** Comparison between the *O. vulgaris* CNS dataset and *A. californica* CNS dataset using BLASTn and tBLASTx.

Most significance Blast E-value	Numbers of *A. californica* ESTs having a blast hit in *O. vulgaris* cDNAs		Numbers of *O. vulgaris* cDNAs having a blast hit in *A. californica* ESTs	
	Blastn	TblastX	Blastn	TblastX
E<1 e^−20^	365	4426	191	3276
1 e^−20^<E<1 e^−15^	287	650	95	614
1 e^−15^<E<1 e^−10^	271	853	190	960
1 e^−10^<E<1 e^−5^	690	1381	662	2840
1 e^−5^<E<1	36006	30943	43356	37751
Total (E<1 e^−5^)	7849		8054(8044)	

The number in each bracket shows the number of genes after removed redundant sequences.

The CNS database of *A. californica* contains two small EST databases: Normalized from Pedal-Pleural ganglia and normalized CNS library (juvenile 1), which provided 40607 sequences after assemble. 3.97% (BLASTn) and 18.00% (tBLASTx) *A. californica* ESTs have a hit in our transcriptome data, while 1.90% (BLASTn) and 12.85% (tBLASTx) of *O. vulgaris* sequences have a significant match in *A. californica*. The low similarity may be due to: 1) the huge diversity of the two species; 2) the CNS database of *A. californica* contains both adult and juvenile CNS tissue (the expression of genes may vary at different growth stages); and 3) though the two parts of *A. californica* database are normalized, some sequences may exist in both databases that significantly downgrade the hit percentage of *O. vulgaris* cDNAs to the *A. californica* ESTs database.

### 5. Comparison with CNS sequence databases of other model organisms

Meanwhile, we chose three different CNS databases belonging to a fish (*Tilapia*) [31], an arthropod (*Schistocerca gregaria*) [32], and an annelid (*Hirudo medicinalis*) [33], all of which were recently published in NCBI, to discuss the diversity among these model organisms in different phylums. We ran BLASTn/tBLASTx searches against the CNS database of fish, dessert locust, and leech. Results are shown in Table 5. With E-value cutoff 1e^−5^, 3544 cDNAs have a hit in Tilapia, the number of hit in other organisms are 5870(Locust) and 6568(Leech). Considering the scale of these CNS databases and the Mollusk species *L. stagnalis* EST database, these hit percentages suggest that *O. vulgaris* has similarity with all organisms except Annelid (*H. medicinalis*). This illustrates that *O. vulgaris* may have both original and evolution characters at the transcriptome level, and this comparison will be helpful for further research in homologous or non-homologous genes of *O. vulgaris*.

**Table 5:** Comparison between *O. vulgaris* dataset and four different species CNS datasets using BLASTn and tBLASTx.

	*S. gregaria*	*Tilapia*	*H. medicinalis*	*L. stagnalis*
EST numbers	34675	10051	87763	10375
Genes after assemble	12709	8180	31332	7712
*O. vulgaris* had a blast hit in ESTs(BLASTn/ tBLASTx, 1e^−5^)	5870	3544	6568	3551
Percentage (%)	46.19	43.32	21.03	46.05

**Figure 4:**
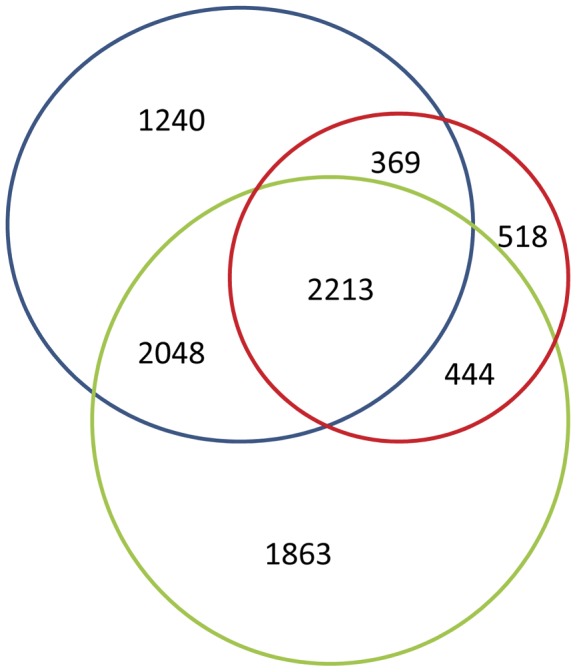
Distribution of BLASTn and tBLASTx hits of *O. vulgaris* sequences in the three organisms (green: *H. medicinalis*, blue: *S. gregaria*, red: *Tilapia*) CNS datasets with E-value threshold of 1e^−5^.

Figure 4 shows the distribution of sequence hits in the locust, fish and leech CNS database. 8,695 sequences match the condition and only 2,213 (25.45%) sequences exist in both 3 databases that are much lower than the same comparison in post studies, which indicate the low percentage of highly conserved genes. This comparison reflects *O. vulgaris*' large number of homologous genes in both vertebrates and invertebrates, indicating that it may be a good model organism for studying neurobiology.

### 6. Phylogenetic analysis of two CNS-related genes

**Figure 5:**
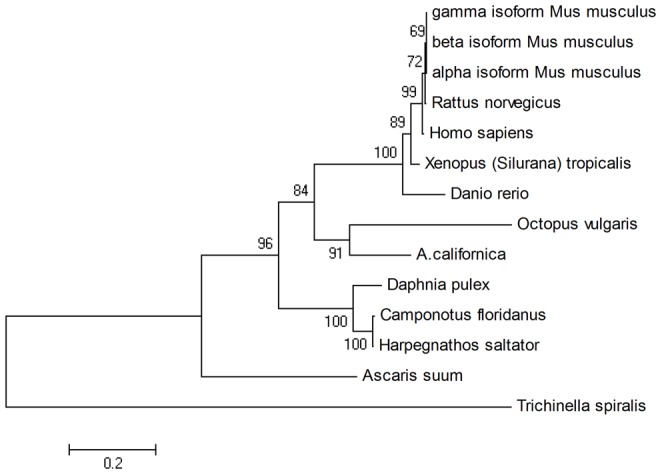
Phylogenetic tree of synaptotagmin-7.

We identified genes related to nervous system functions that existed in the four databases (Leech, Mollusks, Locust and Fish) by applying a Perl script to select the genes meeting the conditions of length (>1,000 bp), E-value (<1e^−10^), and coverage (>30). Then we built the corresponding phylogenetic trees. The tree in Figure 5 is generated by synaptotagmin-7; this protein is a member in the synaptotagmin family believed to be important in the docking and fusion of synaptic vesicles with the plasma membrane, such as neurotransmitter release [34], [35]. We found the sequence of *O. vulgaris* is most related to the Mollusk species *A. californica*. Synaptotagmin-7 widely exists in metazoan and the phylogenetic tree performs well in sorting different categories of animal, indicating that it is a good choice for explaining system evolution. Furthermore, the synaptotagmin-7 of Mollusks is more closely related to arthropods and fish that consistently use the results in comparison to the CNS database between *O. vulgaris* and Locust/Tilapia.

**Figure 6:**
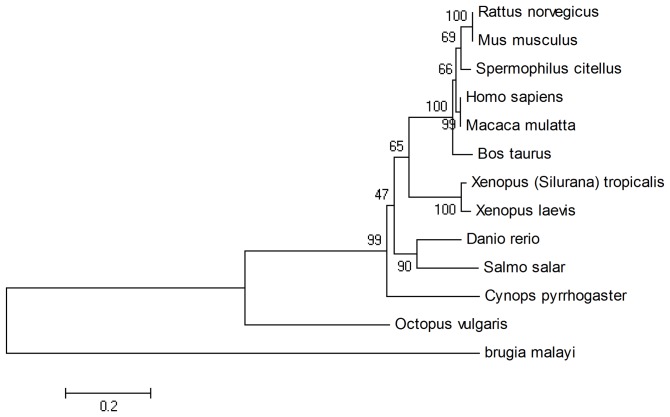
Phylogenetic tree of synaptophysin.

We further chose synaptophysin because they were found in *A. californica* and *L. stagnalis* and are correspond to the phylogenetic tree (Figure 6). Synaptophysin is a protein that acts as a marker for neuroendocrine tumors and quantification of synapses [36]. Even without an ortholog gene in *A. californica* or *L. stagnalis*, the result still reveals the closer relation of *O. vulgaris* to vertebrates.

### 7. A putative vertebrate-like Blood-Brain Barrier in *O. vulgaris*

Cephalopods have a well-developed nervous system, reflected in its precise internal structure and clear healing between ganglions covered with cerebral cortex. In addition, around the CNS there is a cartilaginous skull for protection. These characteristics are similar to vertebrates, indicating that the Cephalopods may be an evolutionary transition to the brain functions of vertebrates. By studying these transitions, we can clearly understand the process of its occurrence. The Blood-Brain Barrier is a kind of internal barrier system related to internal immunity that blocks pathogenic microorganisms and other macromolecules through the blood circulation into brain tissue to maintain the basic stability of the internal environment. It also has the important biological role of maintaining the normal physiological state of the central nervous system [3]. The vertebrate Blood-Brain Barrier has three histological bases: brain microvascular endothelial cells (BMVEC) and junctions between BMVEC, continuous basement membrane around the BMVEC and five different types of neighboring glial cells such as astrocytes, perivascular pericytes, microglia, and surrounding neurons. In addition, to ensure that central neurotransmitters are not able to pass the BBB and maintain stability of neurotransmitter concentrations, BMEVC has a unique enzyme system to inactivate the central neurotransmitters such as monoamine oxidase [37], AAAD, and COMT. We hope to interrogate our CNS dataset and compare it with three other invertebrate model organisms, to find whether Cephalopods have specific gene expression indicative of a vertebrate-like BBB.

The related genes involving junctions between EC, pathways across BBB, and the enzyme barrier system were selected for clarifying whether the molecular foundation of vertebrate-like BBB exists in four invertebrates (*O. vulgaris, A. californica, S. gregaria, H. medicinalis*). First, we focused on all of the important genes involved in tight junctions (TJ) and adherence junctions (AJ) structure such as Claudins [4], [38], Occludin, Junctional adhesion molecules(JAM) [39], Cytoplasmatic proteins [40]–[42] and Cadherins [43], Catenins [44]. At the beginning we searched the gene name annotation result of *O. vulgaris*, two TJ related genes and two AJ related genes were found when E-value<e^−10^. In addition, we carried out a tBLASTn search by using the amino sequences of the TJ and AJ related genes downloaded from NCBI against all CNS Datasets. These results were filtered with a Perl script including a number of strict conditions to ensure accuracy: E-value<e^−10^, the number of amino acids that align to the query sequences >100 and covered more than 80% of subject sequences. After two screening steps, *O. vulgaris* cDNA hit nine of twelve proteins. Considering the alignment features of BLAST and that some proteins have a low conservation between different species, these results indicate that the number of actual proteins will be more than its hit number. This result reveals that *O. vulgaris* has complete BMVEC junctions. After the same filter steps, *A. californica* cDNA hit only five proteins, *S. gregaria* and *H. medicinalis* hit four illustrated that the rest of organisms do not have a significant matching in proteins involving junctions between EC (Table 6).

**Table 6:** List of alignment results of proteins related to TJ and AJ.

		OV	AC	SG	HM
Claudins	Claudins-5	F	F	F	F
Occludin	Occluding	F	F	F	F
Junctional adhesion molecules	JAM-1/JAM-A	T	F	F	F
Cytoplasmatic proteins	ZO-1	T*	F	F	T
	Z0-2	T	F	F	F
	AF-6	F	F	F	F
	Cingulin	T	T	F	F
	Myosin	T*	T	T	T
Cadherins	N-cadherin	T	T	F	F
	Cadherin-5	T	F	F	F
Catenins	α- catenin	T*	T	T	F
	β- catenin	T*	T	T	T

T stands for the sequence eligible the conditions and F stands for the opposite.

* , indicated this gene had been predicted by BLASTx searches in Swissprot/NR protein database. OV*: O. vulgaris*, AC*: A. californica*, SG*: S. gregaria*, HM*: H. medicinalis*.

Besides specific junctions, the elaborate systems for transporting macromolecules and elimination of neurotransmitter are important part of BBB functions [4], [45]. In *O. vulgaris* we found all of the transporters and enzymes that were well-studied exist after the filter steps, except the Caveolae [46], [47]. While in *A. californica* and *S. gregaria*, the miss of glucose transporter-1 [37], [48] reveals the absence of vertebrate-like BBB in these two species. It is impossible for the CNS to function with no energy supply when a physical barrier is established [41], [47]. Another result from exploring enzymes such as monoamine oxidase [37], COMT [49], and AAAD [50] also suggests that *O. vulgaris* has a thorough enzyme system for eliminating the cause of neurotransmitter feedback on the central nervous system. The other organisms are keeping their poor performance in searching the specific transporters and enzymes (Table 7).

**Table 7:** List of the alignment results of proteins involved in specific transporters and enzymes.

		OV	AC	SG	HM
Caveolae	Caveolae-1	F	F	F	F
Glucose transporter	Glucose transporter-1	T*	F	F	T
ATP-binding cassette transporters	P-gp	T*	F	T	T
	Multidrug resistance-associated proteins	T*	T	T	F
organic anion transporter	OAT3	T*	T	T	T
monoamine oxidase	monoamine oxidase A	T*	F	F	F
	monoamine oxidaseC	T*	F	F	F
AAAD	AAAD	T*	T	T	T
COMT	COMT	T*	F	F	F

T stands for the sequence eligible the conditions and F stands for the opposite.

* , indicated this gene had been predicted by BLASTx searches in Swissprot/NR protein database.

OV*: O. vulgaris*, AC*: A. californica*, SG*: S. gregaria*, HM*: H. medicinalis*.

Furthermore,we investigated the profiles of genes involved in TJ signaling pathway, which is considered to responsible for the barrier properties, between *O. vulgaris* and *A. californica* (Figure S2). In default parameters, the number of genes that mapped into the TJ signaling pathway were different (V:47, A:32). Meanwhile this comparative analysis presented three important unique genes in *O. vulgaris:* two transmembrane proteins (JAM and Claudins) and a cytoplasmic TJ accessory protein ZO-1. Claudins are considered to be responsible for permeability restriction in TJ [51] and JAM is involved in various of TJ function such as cell-to-cell adhesion, organizing structure, taking part in the formation of TJ as an integral membrane protein together with claudins [43]. ZO-1 existed as a carboxy-terminal region, which binds to actin and links the TJ to the cytoskeleton, acts as a central organizer of the TJ complex [52]. This result clearly implied that *O. vulgaris* have a more complex and integrated TJ functions than the model organism *A. californica*.

To verify the constitutive expression of the genes corresponding to the BBB structure and function, specific primers were designed based on assembled contigs and quantitative real-time PCR were performed. All of the genes showed a ubiquitous expression in all examined tissues, including brain, liver, heart, gill and muscle (Figure S3). The transcription pattern of these BBB relevant genes had been determined in vertebrates and different kinds of tissues indicated that these genes may not only be involved in BBB but also participated in other physiological functions. For example, ZO-1 mostly expressed in endothelial and epithelial cells forming the TJ assembly, but it still expressed in other tissues not forming TJ that may be involved in signal transduction at cell-cell junctions [43]. Glucose transporter 1 expressed in erythrocytes and also in the endothelial cells of barrier tissues, it also has been identified in muscle, fat and tissues with acute insulin-stimulated glucose transport [53]. Monoamine oxidases are found in neurons, astroglia and also found in the liver, gastrointestinal tract, and placenta that catalyze the oxidative deamination of monoamines [54]. The same results that are similar to above were displayed in other genes related to TJ (myosin [55]), AJ (α-Catenin and β-Catenin [44], Cadherins [56]), specific transporters (P-glyprptein [57], Multidrug resistance-associated protein [4], Organic anion transporter [58]), and enzymes (Catechol-O-methyltransferase [58], Aromatic L-amino acid decarboxylase [59]). These observations not only implied the accuracy of contig assembly, but also demonstrated that as vertebrates, all the target genes can be expressed in different tissues including CNS in *O. vulgaris*.

It is certainly to be noted that the CNS of *O. vulgaris* has a large number of proteins involved in specific junctions, transporters, and enzymes which are definitely indispensable to form an incredible system that may possesses most vertebrate BBB functions. The results in *A. californica* and *S. gregaria* indicate that the species with open vascular system may have a different strategy for protecting the basic stability of the internal environment like *D. melanogaster*. The low hit percentage in *H. medicinalis* demonstrated that although *H. medicinalis* have a similar circulatory system, the loose structure of central nervous system limited the development of vertebrate-like BBB system. Based on the results above, only *O. vulgaris* has the molecular basis of the vertebrate-like BBB, highlighting its use as a model organism for the in-depth study of phylogenetics, structure and function of the BBB.

## Materials and Methods

### 1. Preparation of central nervous system samples

*O. vulgaris* (female) used in this study were obtained from the fish market in Xiamen, Fujian province. Before dissection they were maintained in aquaria. The CNS was dissected under Zeiss steRED Lumar V12 dissection system, including cerebral ganglion, visceral ganglion, pedal ganglion, and part of peripheral nerve. These were isolated and prepared for total RNA extraction.

### 2. RNA isolation, library preparation and sequencing

Using RNAiso Plus (TaKaRa), the total RNA was extracted in accordance with the manufacturer's instructions. The quality of the isolated RNA was checked by electrophoresis on a 1.5% agarose gel and by absorption spectroscopy. The library was constructed using the Mate Pair Library Preparation Kit. We enriched the mRNA by using the magnetic beads which contain Oligo (dT). While filled in the fragmentation buffer, we randomly broke mRNA into small pieces. Random hexamers were used for the synthesis of the first strand of cDNA. The complementary strand was synthesized. With several steps of purification, adaptor addition and cDNA length selection, this library was used for sequencing on an Illumina HiSeq 2000. A paired-end Solexa sequencing strategy was used for sequencing to better understand the gene expression profile and for convenient assembly of the entire transcriptome *de novo*.

### 3. Transcript assembly and annotation

Transcripts were assembled using VELVET software, 59,859 contigs were generated and 31,909 contigs with length ≥200 bp were submitted to the TSA (Transcriptome Shotgun Assembly Sequences Database) (Genbank TSA Acc. Number: JR435555–JR467463). Contigs with length <200 bp were deposited in table S2. To annotate these sequences we aligned them using the Swissprot, NR protein databases and NT nucleotide database. Sequences producing an E-value<1e^−5^ were considered a hit. Read number was determined based on the relationship between contig length and contig coverage. Functional GO annotations were performed using GOminer and KEGG pathway analysis was carried out using KASS (KEGG Automatic Annotation Server) with default parameters and 5 model organisms in vertebrate (*Homo sapiens, Mus musculus, Gallus gallus, Xenopus laevis, Danio rerio*) that were chosen as genes data set.

### 4. Comparison to post published CNS databases and phylogenetic analysis

BLASTn and tBLASTx were carried out for testing the similarity between *O. vulgaris* and other model organisms' CNS database (*A. californica, L. stagnalis, S. gregaria, H. medicinalis, Tilapia)* at both the nucleotide and amino acid level with a threshold E-value less than 1e^−5^. Phylogenetic analysis was carried out using the sequences that had been annotated and met the conditions of sequence length (>1,000 bp), E-value (<1e^−10^) and coverage (>30). Then the sequences were selected for building the phylogenetic tree using the neighbor-joining method.

### 5. Identification of genes involved in the vertebrate-like Blood-Brain Barrier

To identify the putative genes involved in the vertebrate-like Blood-Brain Barrier, the sequences from this study and published invertebrate CNS database were screened using confirmed protein sequences in NCBI, which had been demonstrated to have involved specific structures or functions of vertebrate BBB. tBLASTn was used for scanning and a Perl script for screening the blast results with E-value<1e^−10^. The number of amino acids that align to the query sequences>100 and covered more than 80% of subject sequence ensure that the alignment between sequences are of global significance.

### 6. Gene verification by Real-time PCR

The expression of 14 target genes related to Blood-Brain Barrier were Validated by quantitative Real-time PCR (qRT-PCR) using in ABI 7900HT Real-time Detection System (Applied Biosystems, USA) with SYBR® Premix DimerEraser (TaKaRa, Japan), and the house-keeping gene β-actin was used as internal standard gene. Primers for all target genes as well asβ-actin gene were listed in Table 8.

**Table 8:** Primers for 14 target genes and β-actin gene used for Real-time PCR.

Gene (contig number)	Primer	Primer sequence (5′-3′)
myosin	myosin_F36	GGAAAGTTCAGTGTTCTCCGTCAT
(NODE_94559)	myosin_R170	TACGAAACAGGTTAGACTCCAAAG
glucose transporter	glu-tra_F28	TGCGTGAGTGACGGTGGAGATGTG
(NODE_48440)	glu-tra_R199	ACATCCACCTCTGCCAGCAGATG
monoamino oxidase (MONO)	MONO_F1170	GGGCGGAAGAACAATACAGTGGA
(NODE_2713)	MONO_R1453	AAAGAAGGTCTCATCGAAAGGAC
tight junction protein 1 (ZO-1)	ZO_F55	TCCTTCTGGTAGAGCACCTTTT
(NODE_84454)	ZO_R210	CTGGAAACAGACCACGAAAAAT
alpha-catenin	A-catenin_F640	GGCGACAATGATGCTGCTAACTT
(NODE_17600)	A-catenin_R798	AGGCTGTCACTAGACACTCCACCA
beta-catenin	B-catenin_F1262	GGACTACTTCCAGAGTACTTAAAGT
(NODE_2995)	B-catenin_R1408	TTCTCAGAGTCCAGAGGCAGTTAT
Catechol-O-methyltransferase (COMT)	COMT_F137	ATGGGCAGGTCAAGTCCTCAAGAT
(NODE_96469)	COMT_R348	TGATGGTGATTTTCTTGCCATGTG
Aromatic L-amino acid decarboxylase (AAAD)	AAAD_F222	GCCTCATACCATTTTTCGTTTGTG
(NODE_49080)	AAAD_R395	CGCCATTCAGTAGGGGTCGGTATT
Multidrug resistance-associated protein (MRS)	MRS_F217	GAGGGAATCAGTTTCCGCATCAAC
(NODE_85752)	MRS_R430	CAGATGAACAAATATGGTCGGCACT
P-gp	Pgp_F193	TGCTAATGACGCTTGGATGTTTCT
(NODE_10911)	Pgp_R360	CGTTATGATACGCTTGTTGGTGAA
cingulin	cingulin_F590	ATTCTTCCTTCTCACCAGCCTCAC
(NODE_5285)	cingulin_R771	AGAAATCACTCGGTTAAACACTC
Junctional adhesion molecule-1 (JAM)	JAM_F144	CCTTCAACAGGCTCACTCACTTCT
(NODE_6101)	JAM_R375	CTCCCAAAGTTCAGTGGAGATACA
Organic anion transporter (OAT)	OAT_F122	CATAGGCTTAGCAGAGTTTCCAG
(NODE_79656)	OAT_R292	GTCAAAGCGATAGTAAACCCAAT
N-cadherins	cadherins_F300	TGACCCTACCATTCCGTATCACAA
(NODE_4866)	cadherins_R443	CCACCACATTGGCAGTGTTGTTC
β-actin	β-actin_F1	TGATGGCCAAGTTATCACCA
	β-actin_R1	TGGTCTCATGGATACCAGCA

Total RNA was prepared from the tissues (brain, liver, heart, gill and muscle), and the first-strand cDNA synthesis was the same as described in section 1. PCR amplifications were performed in triplicate wells under following conditions: initial denaturation at 95°C for 30 s followed by 40 cycles of 95°C 5 s, 55°C for 30 s and 72°C 10 s. Dissociation analysis of amplification products was performed at the end of the PCR reaction. After the PCR program, data were analyzed with ABI 7900HT SDS software 2.3 (Applied Biosystems, USA). To maintain consistency, the baseline was set automatically by the software. The cycle threshold values were converted into the equivalent target amount using the external calibration curves (correlation coefficient >0.99), which were based on 15 standard targets of recombinant PMD19-T containing 14 target genes and β-actin gene fragment, respectively. All data were given in terms of relative mRNA expressed as means±SE.

## Supporting Information

Figure S1 **ORF distribution of contigs obtained from** ***O. vulgaris*** **central nervous system (CNS) transcriptome library.**
(TIF)

Figure S2 **The tight junction pathway in** ***O. vulgaris*** **and** ***A. californica*** **.**
(RAR)

Figure S3 **Tissue distribution of 14 BBB-related genes.**
(RAR)

Table S1 **Summary table for assembly.**
(DOCX)

Table S2 **Contigs with length less than 200 bp.**
(TXT)
