# Supplementary figures and images for: Transcriptome Analysis of the Octopus vulgaris Central Nervous System
Source: PLoS One. 2012 Jun 29;7(6):e40320. doi: 10.1371/journal.pone.0040320 (PMC3387010; doi:10.1371/journal.pone.0040320)

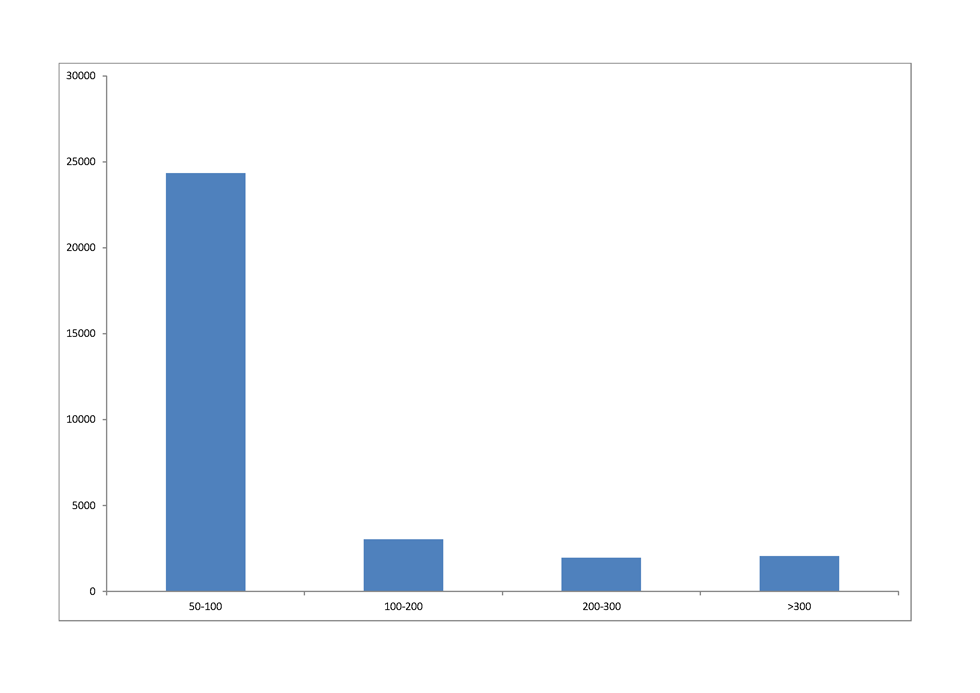

Supplement: Figure S1 — ORF distribution of contigs obtained from O. vulgaris central nervous system (CNS) transcriptome library. (TIF) [file pone.0040320.s001.tif]
