# Supplementary material for: Transcriptome Analysis of the Octopus vulgaris Central Nervous System
Source: PLoS One. 2012 Jun 29;7(6):e40320. doi: 10.1371/journal.pone.0040320 (PMC3387010; doi:10.1371/journal.pone.0040320)
Supplement: Table S1 — Summary table for assembly. (DOCX) [file pone.0040320.s004.docx]

| K | 31 |
| --- | --- |
| no. of reads used for assembly | N/A |
| no. and % reads not used or assembly | N/A |
| total no .of contigs | 59859 |
| total length of contigs | 19,442,767bp |
| avg length of contigs | 324.8bp |
| longest contig | 7624 |
| no. of contigs equal or greater 100 bp | 54123 |
| total length contigs equal or greater 100 bp | 18,913,114bp |
| avg length of contigs equal or greater 100 bp | 349.5bp |
| N50 for contigs equal or greater 100 bp | 466 |
